# Kinetic Analysis Reveals the Role of Secondary Nucleation in Regenerated Silk Fibroin Self-Assembly

**DOI:** 10.1021/acs.biomac.2c01479

**Published:** 2023-03-16

**Authors:** Ayaka Kamada, Zenon Toprakcioglu, Tuomas P. J. Knowles

**Affiliations:** † Department of Chemistry, University of Cambridge, Lensfield Road, Cambridge CB2 1EW, U.K.; ‡ Cavendish Laboratory, University of Cambridge, Cambridge CB3 0FE, U.K.

## Abstract

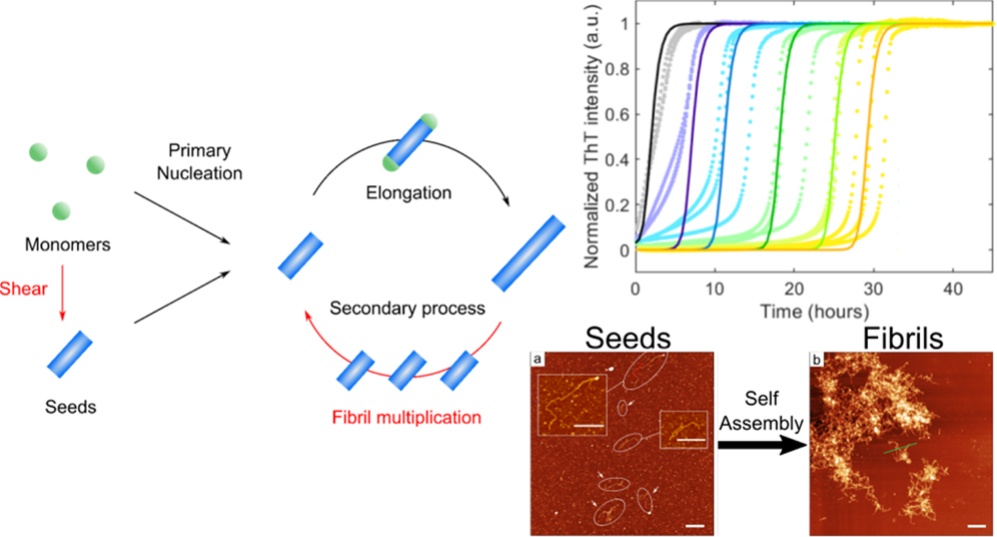

Silk proteins obtained from the *Bombyx mori* silkworm have been extensively studied due to their remarkable mechanical properties. One of the major structural components of this complex material is silk fibroin, which can be isolated and processed further in vitro to form artificial functional materials. Due to the excellent biocompatibility and rich self-assembly behavior, there has been sustained interest in such materials formed through the assembly of regenerated silk fibroin feedstocks. The molecular mechanisms by which the soluble regenerated fibroin molecules self-assemble into protein nanofibrils remain, however, largely unknown. Here, we use the framework of chemical kinetics to connect macroscopic measurements of regenerated silk fibroin self-assembly to the underlying microscopic mechanisms. Our results reveal that the aggregation of regenerated silk fibroin is dominated by a nonclassical secondary nucleation processes, where the formation of new fibrils is catalyzed by the existing aggregates in an autocatalytic manner. Such secondary nucleation pathways were originally discovered in the context of polymerization of disease-associated proteins, but the present results demonstrate that this pathway can also occur in functional assembly. Furthermore, our results show that shear flow induces the formation of nuclei, which subsequently accelerate the process of aggregation through an autocatalytic amplification driven by the secondary nucleation pathway. Taken together, these results allow us to identify the parameters governing the kinetics of regenerated silk fibroin self-assembly and expand our current understanding of the spinning of bioinspired protein-based fibers, which have a wide range of applications in materials science.

## Introduction

Native silk from the silkworm *Bombyx mori* consists predominantly of two proteins, sericin and fibroin. The latter is one of the most common protein-based feedstocks used in biomaterial science,^1−4^ and even though extraction of this protein is quite crude, regenerated silk fibroin (RSF) has been extensively studied in biomedically related applications.^5^ Due to its abundance and biocompatibility as well as its remarkable mechanical properties, such as high strength and flexibility, this material is a promising candidate for a wide range of applications, including biomedicine,^6^ bioelectronics,^7^ and microphotonics systems.^8^ Macroscopic materials formed from regenerated silk feedstocks consist of supramolecular nanofibrillar structures self-assembled from monomeric protein-building blocks.^9,10^ During self-assembly, RSF undergoes a conformational change from random coil to form ordered β-sheets, which are stabilized through a dense intermolecular hydrogen-bonding network. This network contributes in a central manner toward the remarkable mechanical performance of regenerated silk fibroin materials.^11−17^ The self-assembly of RSF is a complex process that can be induced and modulated by various factors including chemical environment, ion concentration, pH and solvent conditions^18,19^ as well as physical parameters such as temperature^20^ and hydrodynamic shear forces.^21−24^ However, the molecular mechanism of RSF aggregation, and in particular, the effect that hydrodynamic shear has on the fibrillization of this protein remains largely unresolved.^25,26^

To date, regenerated silk fibroin self-assembly has been studied using rheology, where the gelation process gives an indication on the quantity of fibrils formed.^27−32^ However, with such approaches, self-assembly is typically observed through its effects on the mechanical parameters, including storage modulus or viscosity. Moreover, several techniques including circular dichroism,^33−36^ infrared spectroscopy,^37−40^ and X-ray scattering^41^ have been used to monitor the formation of β-sheets during self-assembly. An orthogonal approach toward monitoring fibrillar self-assembly that allows the kinetics of this process to be tracked in real time exploits the use of intrinsic fluorescence from the aromatic amino acids such as tryptophan.^42^ Moreover, the use of extrinsic fluorophores such as Thioflavin T (ThT), which has the propensity to increase its quantum yield upon binding to β-sheet rich fibrils, have proven invaluable in monitoring aggregation reactions.^43,44^ By coupling such approaches to the framework of chemical kinetics, recent work has been able to discover the assembly mechanisms of a number of fibrillar self-assembly systems, including amyloid fibrils.^45−49^

Here, we apply the framework of chemical kinetics to investigate the self-assembly of regenerated silk fibroin (RSF). This approach provides a means of sampling a wide range of initial conditions in a systematic manner and connecting experimental measurements to molecular mechanisms, allowing us to elucidate the relative importance of different kinetic processes involved in RSF self-assembly. Our results demonstrate that the self-assembly of RSF is strongly dominated by nonclassical secondary nucleation process, in which the formation of new nuclei is strongly catalyzed by existing aggregates. Such secondary pathways have been initially discovered in the context of pathological protein polymerization,^50^ including sickle hemoglobin^51^ and the amyloid-β peptide associated with Alzheimer’s disease,^52^ but the present results demonstrate that this process can also take place in the assembly of functional materials without any association with a disease state. We further applied this approach to investigate the effect of hydrodynamic shear on RSF self-assembly. Through this approach, we demonstrated that the aggregates formed by shear promote fibrilization through the secondary process. These findings shed light on the molecular mechanisms of regenerated silk fibroin self-assembly and provide new opportunities to fabricate next-generation RSF-based materials.

## Materials and Methods

### Preparation of Protein Solutions (Regenerated Silk Fibroin and Insulin)

*B. mori* silk cocoons (Mindsets (U.K.) Limited) were used to extract the silk fibroin protein using a previously established protocol.^53^ Initially, the cocoons were cut into pieces and placed in a beaker containing a solution of 0.02 M sodium carbonate (Sigma-Aldrich). This was then boiled for 30 min, ensuring that the sericin that was present within the silk fibers dissolved while the insoluble fibroin remained in the beaker. The fibroin was then removed from the beaker, rinsed with cold water three times, and left in a fume hood to dry out.

A 9.3 M lithium bromide (Sigma-Aldrich) solution was prepared and added to the dried silk fibroin in a 1:4 ratio of silk fibroin to lithium bromide. The mixture was heated to 60 °C and left for 4 h, resulting in a translucent silk fibroin solution. Lithium bromide was removed from the silk solution through dialysis against ultrapure water using 3 kDa dialysis membrane at 4 °C. The water was changed a total of five times in 48 h. Finally, the silk fibroin solution was collected and then centrifuged at 9000 rpm at 4 °C for 20 min to remove small impurities. The process was repeated twice, and the final solution was stored at 4 °C.

As a comparison to RSF, insulin (from bovine pancreas, Sigma Aldrich) was also sheared, and its aggregation kinetics were studied. Fresh insulin solution was prepared by dissolving insulin powder in pH 1.4 hydrochloric acid.

### Kinetic Curve Acquisition Using Thioflavin T

The aggregation kinetics of RSF was studied by means of a Thioflavin T (ThT) fluorescence assay. ThT has the ability to increase its quantum yield upon binding to β-sheet structures and is thus commonly used for studying the formation of amyloid fibrils. The protein solutions were loaded into a 96 well microplate (Corning 3881) and mixed with ThT. For each cell, 100 μL of protein solution and 1 μL of 10 mM ThT solution were added. The plate was sealed with aluminum tape and incubated at 37 °C for RSF and 50 °C for insulin in the plate reader (FLUOstar Omega microplate reader, BMG Labtech). The fluorescence intensity during the incubation was measured with filters at 440 nm excitation and 480 nm emission. All measurements were performed without agitation.

For the experiments involving seeds, pre-made aggregates were used, which were obtained from incubating 1 mg/mL RSF solutions for one month at 37 °C. The seeds solution was sonicated for 2 min using an ultrasonic homogenizer (Bandelin HD4200 with 10% power, 0.5 s pulse per second) and added to the monomer solution right before the incubation.

### Shearing of Proteins

To study the effect of shear by Couette flow, a rheometric setup (ARES controlled strain rheometer, Rheometric Scientific) was used. 400 μL of protein solution was placed on the rheometer and sheared using 1.003 radians angled cone plate with 25 mm diameter. The shear rate was fixed at either 100 or 500 s^–1^, and samples were collected immediately after the certain time of shearing had finished. The temperature of the bottom plater was set at 10 °C during the shearing using a water jacket, and all samples were kept in an ice bath before and after the shearing to minimize protein self-assembly during the transfer of the solutions from the rheometer to the plate reader.

### Kinetic Analysis

The obtained ThT intensity curve as a function of time was first normalized. It should be noted that RSF monomer contains a small amount of β-sheet, leading to the initial absolute fluorescence intensity corresponding to ca. 10% of the absolute fluorescence signal of the final aggregate mass. However, the initial intensity was set to zero after the normalization, assuming that the initially present β-sheets are not aggregates. The seeded data were normalized with respect to the amount of fibrils present in solution, i.e., a 10 w/w% seeded solution starts at a normalized intensity of 0.1 rather than at 0. The normalized kinetic curve was then analyzed using Amylofit. The data were first fitted using a secondary nucleation dominant model described by the following differential equations
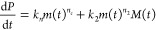

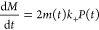
where *P* and *M* refer to the aggregate number concentration and the aggregate mass concentration, respectively. The primary nucleation rate *k*_*n*_, secondary nucleation rate *k*_2_, and elongation rate *k*_+_ were fitted globally, whereas the primary nucleus size *n*_*c*_ and secondary nucleus size *n*_2_ were fixed at 1 and 2, respectively. The initial monomer concentration *m*(0) was set to 1 μM for 1 mg/mL RSF, assuming that the molecular weight of RSF is ca. 100 kDa.^50^ Additionally, the kinetic data were fitted using a primary nucleation and elongation model
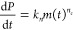

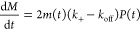
The primary nucleation rate *k*_*n*_ and elongation rate *k*_+_ were fitted globally, whereas the primary nucleus size *n*_*c*_ and the depolymerization rate *k*_off_ were fixed at 1 and 10^–15^ h^–1^, respectively. For both models, the initial aggregate mass *M*_0_ and the initial aggregate number concentration *P*_0_ were set as free fitting parameters. For the seeded kinetics (1 mg/mL RSF with 0–10 w/w% seeds), the initial aggregate mass *M*_0_ obtained from the fitting was correlated with the actual seed concentration (0–10 w/w%). This correlation was then used to calculate the seed concentration formed by shear using the initial aggregate mass *M*_0_ obtained from the fitting of the sheared RSF kinetics.

### Atomic Force Microscopy (AFM)

The aggregates formed due to shear and subsequent incubation at 37 °C were characterized through atomic force microscopy (Park NX10, Park Systems). The sheared samples were collected from the rheometer and immediately diluted 100 times in water. The incubated samples were also diluted 100 times in water. A 10 μL aliquot of the diluted solution was then placed on a freshly cleaved mica and incubated at room temperature for 5 min. The mica was gently washed with Milli-Q water and dried with compressed nitrogen gas. The images were obtained using a noncontact mode immediately after the sample preparation.

#### Fourier-Transform Infrared Spectroscopy (FTIR)

The conformational changes of regenerated silk fibroin were conducted using an FTIR equinox 55 spectrometer (Bruker). The samples were first loaded onto the FTIR sample holder and were analyzed by subtracting a water reference. Additionally, a carbon dioxide atmospheric compensation was performed by subtracting this from the FTIR spectra. All FTIR measurements were conducted at room temperature.

## Results

### Kinetic Analysis Reveals Secondary Processes in Regenerated Silk Fibroin Self-Assembly

**Figure 1:**
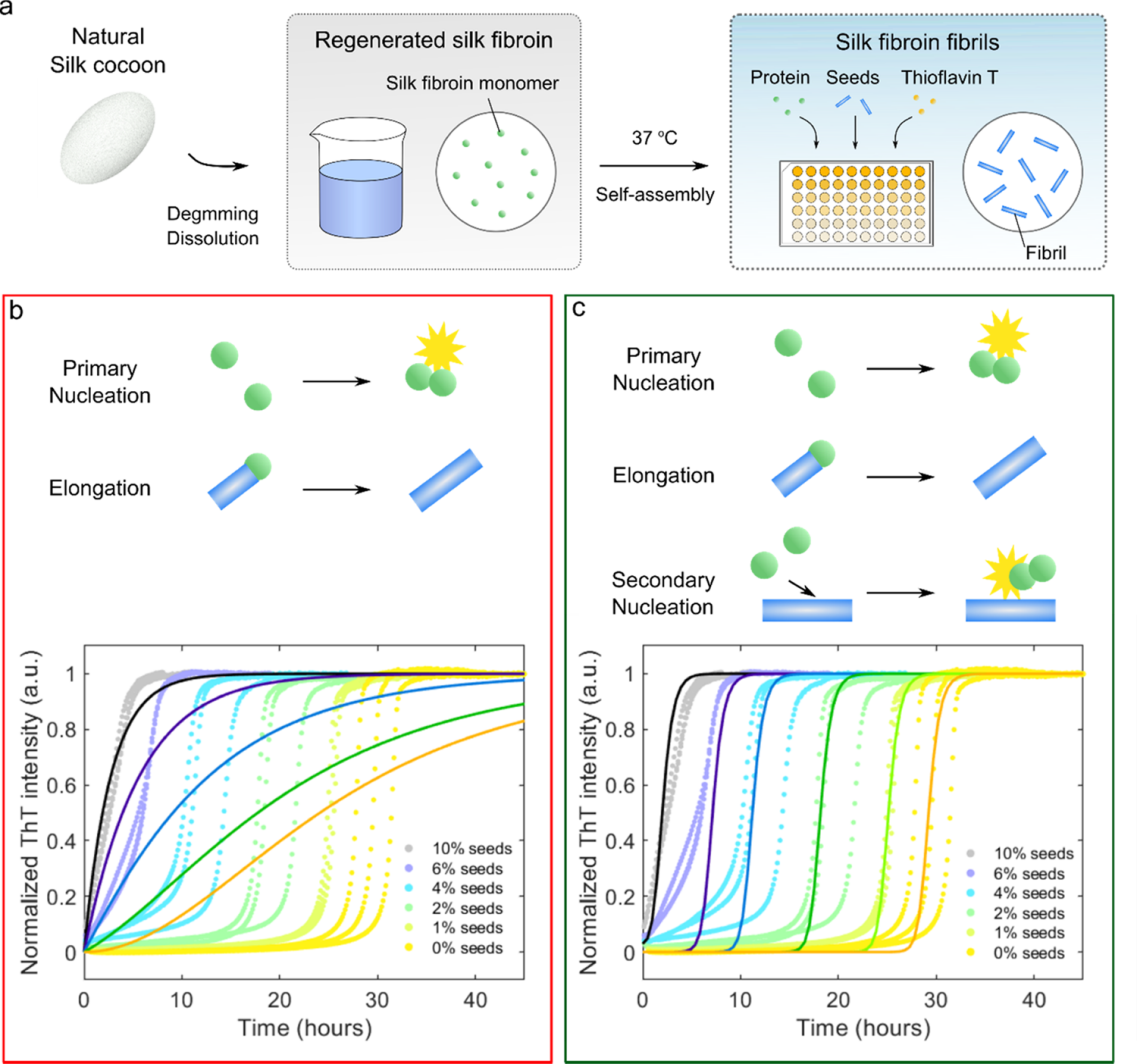
Seeded aggregation kinetics of regenerated silk fibroin. (a) Schematic illustration of the experimental approach. The kinetic behavior of regenerated silk fibroin (RSF) was studied using Thioflavin T (ThT). (b, c) Normalized ThT fluorescence intensity as a function of time for 1 mg/mL RSF with a seed concentration of 0–10 w/w%, incubated at 37 °C. The plots with the same color indicate replicates. The fits to each data set are shown as the solid lines. The solid lines show the best fitting for a primary nucleation and elongation model (b) and for a primary nucleation, elongation, and secondary nucleation model (c).

To discover the molecular mechanisms underlying regenerated silk fibroin assembly (RSF), we used chemical kinetics to analyze kinetic measurements of the assembly process. This approach has successfully been used to solve the mechanism of aggregation of a number of filamentous systems associated with disease, and we apply this strategy here for the first time to the process of regenerated silk fibroin assembly. To this effect, a silk fibroin monomer solution was regenerated from natural feedstocks of silk cocoons from the silkworm *B. mori*, using a previously established protocol.^53^ This resulted in the formation of a regenerated silk fibroin system, RSF, which is used in the generation of artificial functional materials. This solution was then incubated at 37 °C to induce the self-assembly, while the fluorescence signal from an extrinsic fluorophore, ThT, was monitored using a microplate reader. During the incubation, RSF changes its conformation from random coil to β-sheet, leading to an increase of ThT fluorescence intensity.^33,42,54−56^ To investigate the kinetic processes involved in RSF self-assembly, we first examined the effect of preformed seed aggregates on the monomeric protein. Such seeding experiments allow us to control initial concentration of aggregate mass, thus enabling to elucidate the relative importance of the two fundamental processes responsible for generating new aggregates: classical primary nucleation, which converts monomers directly into aggregates, and nonclassical secondary processes, which generate new aggregates in processes which facilitated by the presence of existing aggregates.^52^ The formation of the seed structures was carried out by incubating 1 mg/mL RSF solution at 37 °C for a week to induce complete self-assembly, followed by ultrasonication treatment to generate fragmented fibrils. The obtained seeds were then added to a fresh 1 mg/mL monomer solution with different initial concentrations. We found that the addition of only 1 w/w% seeds bypassed primary nucleation entirely, leading to a significantly shorter lag time as compared to the unseeded sample. Crucially, however, the shape of the kinetic profiles maintained an initially convex shape, a characteristic that indicates the formation of new aggreges through an active nucleation process. This high sensitivity of RSF aggregation to such small seed amounts suggests that there is a strong self-replicating secondary process where the formation of new fibrils is catalyzed by the existing fibrils—the signature of secondary processes. To further corroborate the presence of the secondary processes, the kinetic data were fitted to microscopic rate laws describing filament formation through either primary or secondary nucleation using the software platform Amylofit.^45^ The results show that the best fit for the primary nucleation and elongation model was not capable of replicating the experimental data (Figure 1b, solid lines). However, the addition of secondary nucleation to this model leads to a good global description of the entire data set over different seed concentrations (Figure 1c, see Methods for parameter details used for fitting).

### Role of Hydrodynamic Shear on the Primary Nucleation Process

**Figure 2:**
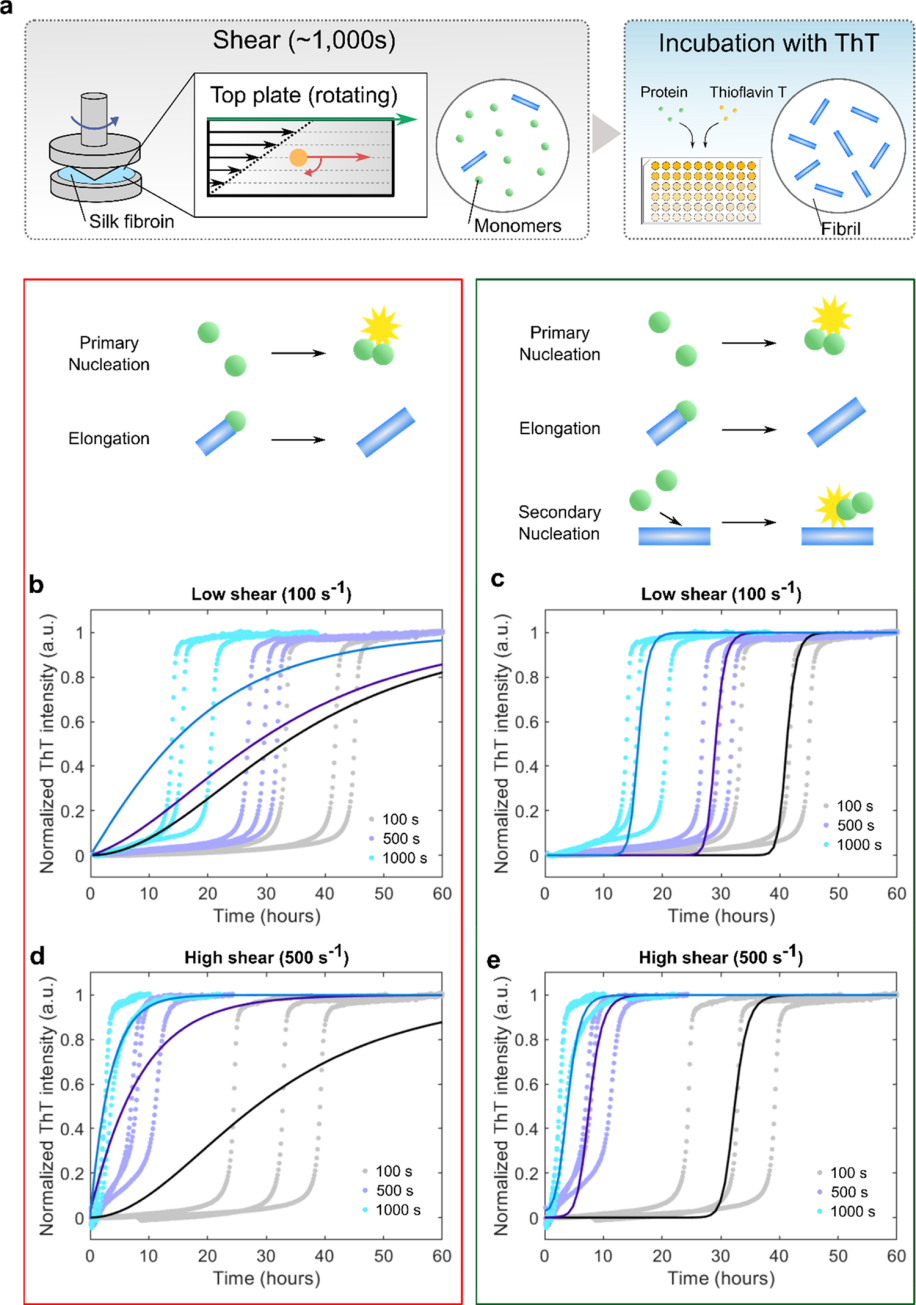
Aggregation kinetics of regenerated silk fibroin following exposure to a shear force. (a) Schematic illustration of the experimental setup, where regenerated silk fibroin was mechanically sheared through the use of a rheometer and subsequently incubated with ThT to monitor the increase of fluorescence intensity. (b–e) ThT fluorescence intensity as a function of time for a 2.5 mg/mL regenerated silk fibroin solution, sheared with different shear rates, 100 s^–1^ (b, c) and 500 s^–1^ (d, e). (b, d) Best fitting using primary nucleation and elongation model. (c, e) Best fitting using primary nucleation, elongation, and secondary nucleation model.

**Figure 3:**
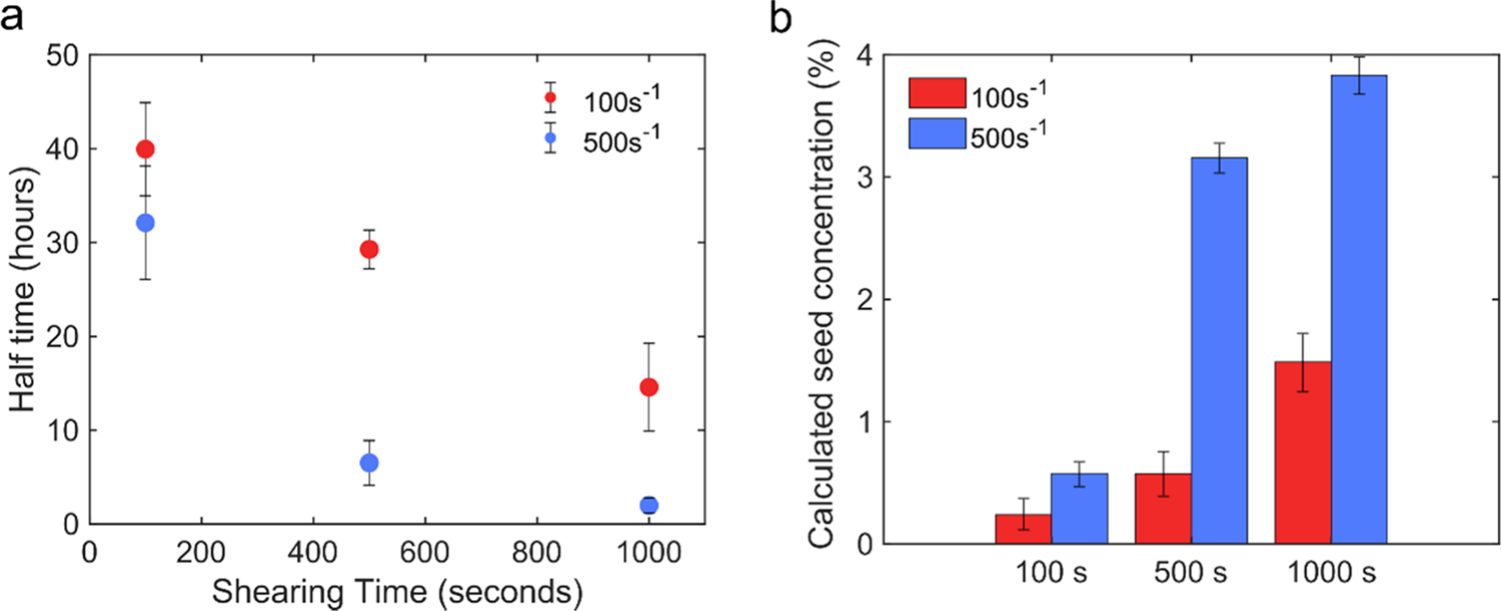
Estimated amount of seeds formed by shear. (a) Plot of aggregation half-time as a function of shearing time for different shear rates (100 and 500 s^–1^). (b) Estimated amount of seeds (% to total protein weight) formed by shear calculated through fitting with Amylofit.

We further expanded the kinetic experiments to investigate how hydrodynamic shear affects the fibrillization process of RSF. This was achieved by flow-shearing monomeric protein solution under various shear conditions and subsequently incubating with ThT using a plate reader (Figure 2a). A rheometer with a cone and plate geometry was used to introduce a Couette flow, where the shear rate was chosen at either 100 or 500 s^–1^. The kinetic data for RSF treated with the shear rate of 100 and 500 s^–1^ are shown in Figure 2b–e, respectively. For each shear rate, a shear duration ranging from 100 to 1000 s was used. It was observed that with longer shear duration, faster aggregation kinetics were detected, which is confirmed by the shift of the typical sigmoidal kinetic curve toward shorter half-times (Figure 3a). While this effect was observed for both shear rates, higher shear rates (500 s^–1^) promote faster aggregation when compared to lower shear rates (100 s^–1^). Furthermore, a similar effect on aggregation kinetics following shear flow was observed for lower RSF concentrations, 0.5 mg/mL (see Supporting Figure S1). Interestingly, this level of enhancement of the primary nucleation rate of regenerated silk fibroin by shear is significantly higher than that observed for other amyloid-forming proteins, including insulin (Supporting Figure S2).

It was observed that the maximal gradient of the kinetic curves during the growth phase is approximately the same for all samples of sheared RSF, independently of the shearing time. However, the lag time was significantly reduced for increased shearing time. This behavior is characteristic of systems polymerizing under the action of secondary nucleation in the presence of seeds.^45^ Indeed, this behavior was strikingly similar to the seeded data shown in Figure 1 and suggested that shear promotes the formation of primary nuclei capable of further growth by elongation and multiplication through secondary pathways.

To gain further insights into the molecular-level steps involved in this process, we fitted the kinetic data to a series of molecular rate laws to test specific mechanisms of assembly. The fitting results (Figure 2) demonstrate that the kinetic behavior of sheared RSF was not captured by a primary nucleation and elongation model alone. However, when the secondary nucleation was added to the primary nucleation and elongation model, it was possible to fit the experimental data for both samples sheared at the high shear rate (500 s^–1^) and the low shear rate (100 s^–1^). The seed concentration estimated from the fitting was 3.8 w/w% for the sample sheared at the high shear rate (500 s^–1^, 1000 s) and 1.5 w/w% for the sample sheared at low shear rate (100 s^–1^, 1000 s, Figure 3b).

**Figure 4:**
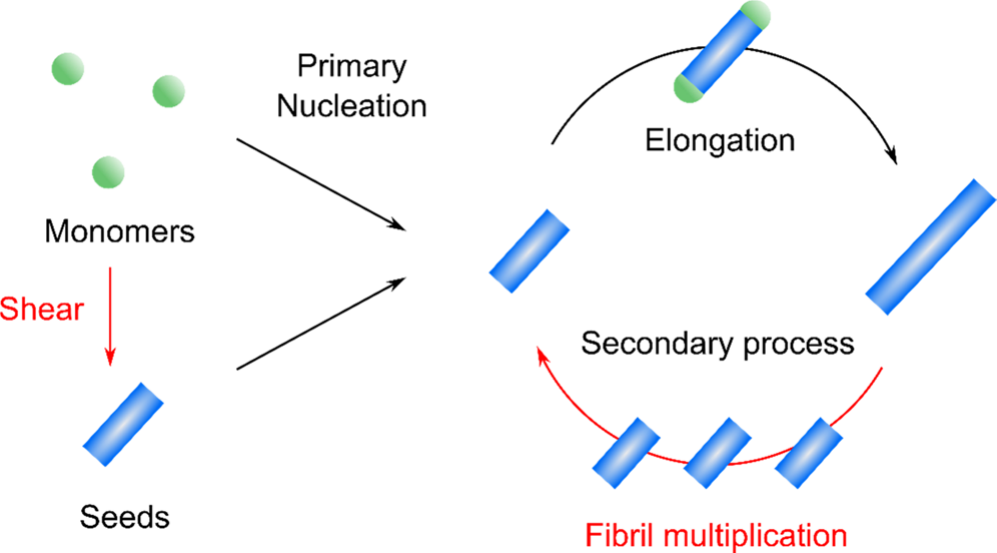
Schematic illustration of secondary processes and the effect of shear on regenerated silk fibroin self-assembly. Shear force can induce the formation of seeds, which bypass the primary nucleation and thus accelerate the fibrillization via secondary pathways.

Taken together, these data show that the basic molecular architecture of the mechanism by which shear accelerates the formation of RSF nanofibrils is an autocatalytic cycle where the existing aggregates catalyze the formation of new fibrils (Figure 4). As seen in the seeded experiment (Figure 1), the self-assembly of RSF is dominated by a secondary process, where the formation of new fibrils is catalyzed by the formed nuclei. Such secondary processes include the secondary nucleation pathway by which the monomers form oligomers at the surface of the existing fibrils. When shear is applied to RSF solution, some monomers are transformed into seeds, which are relatively small amount by weight (only up to 3.8 w/w% of total protein even after long shearing at a high shear rate). However, due to the strong dependency of RSF to self-assemble via a secondary process, the seeds formed due to shear can significantly accelerate fibril formation and thus lead to faster formation of RSF nanofibrils.

Finally, the structural transitions undergone by the sheared silk fibroin were determined by conducting FTIR measurements. The monomeric regenerated silk fibroin solution (blue curve) has peaks at 1650 and 1545 cm^–1^ that are characteristic of random coil conformation and correspond to the amide I and II bands, respectively (Supporting Figure S3). The sample was then sheared at a shear rate of 500 s^–1^ for 100 s. As can be seen from the spectra, the amide I and amide II peaks have slightly shifted toward lower wavenumber, suggesting partial aggregation (or the formation of some seeds) has occurred (green curve). An FTIR spectrum following a 72 h incubation at 37 °C was obtained (red curve). Both amide I and II peaks have further shifted to around 1630 and 1520 cm^–1^, respectively, confirming the structural transition from random coil to β sheets. These results are all summarized in Supporting Figure S3.

### Morphology of Regenerated Silk Fibroin Aggregates Formed by Hydrodynamic Shear

**Figure 5:**
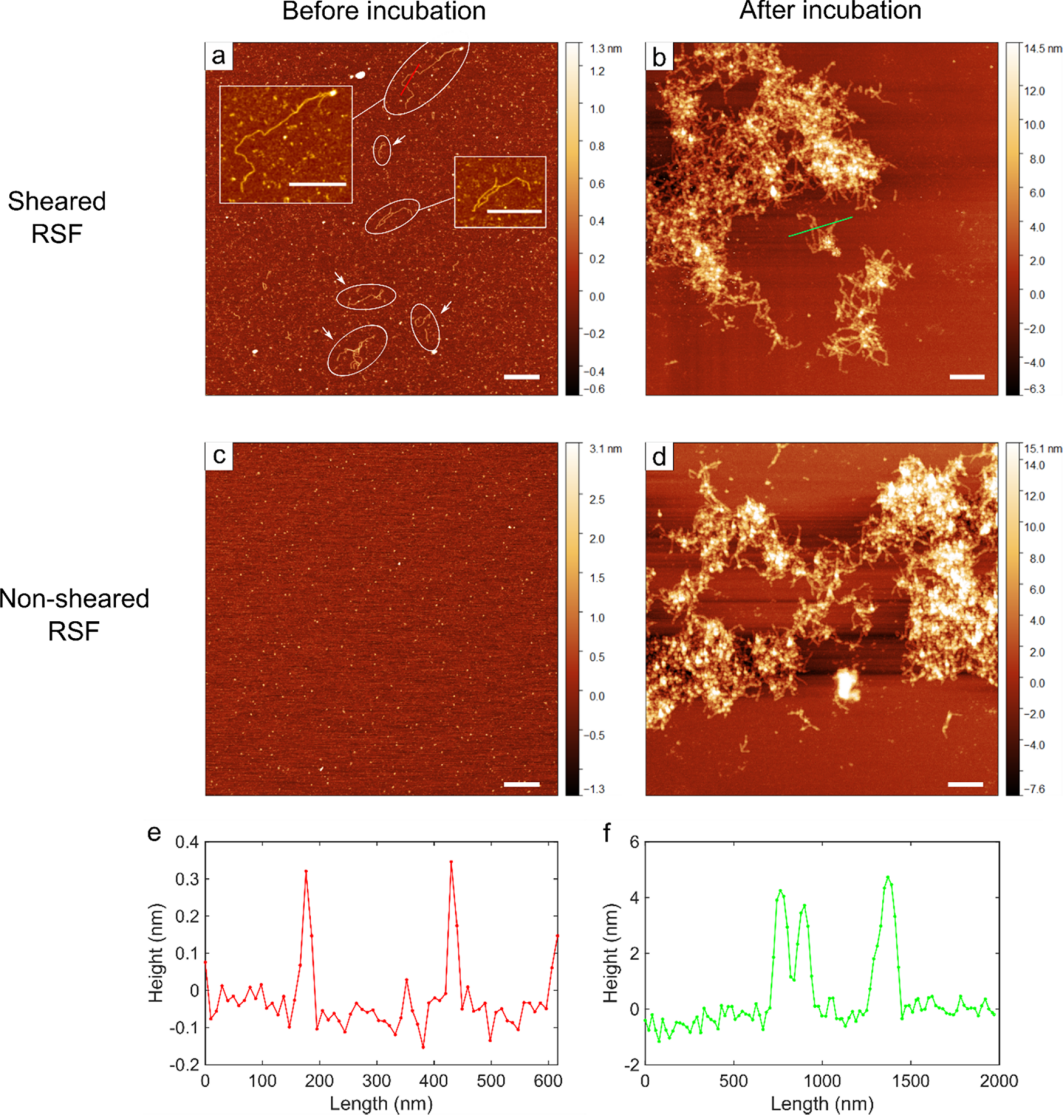
Morphology of regenerated silk fibroin fibrils under no-shear and high shear conditions. (a, b) AFM micrographs of regenerated silk fibroin sheared for 500 seconds with a shear rate of 500 s^–1^ using the rheometric setup with a cone and plate geometry. The images were taken before (a) and after (b) the self-assembly through incubation at 37 °C for 72 h. (c, d) AFM micrographs of the protein without shearing. The images were taken before (c) and after (d) self-assembly through incubation at 37 °C for 72 h. All scale bars are 1 μm. (e, f) Height profiles of fibrils formed due to shear (e) and fibrils formed after incubation (f).

To further investigate the morphologies of the aggregates induced due to shear, AFM micrographs were taken for the RSF samples sheared at 500 s^–1^ for 500 s. The images revealed that following shearing, a small amount of filaments could be observed (Figure 5a), whereas such filaments were not present in the absence of shear (Figure 5c). Furthermore, both no nsheared and sheared RSF solutions were incubated at 37 °C for 72 h to induce self-assembly and their morphologies were again imaged. It was found that the morphologies of the fibrils formed following incubation do not show a significant difference between the sheared and nonsheared samples (Figure 5b,d). The height of fibrils formed after incubation is ∼4 nm (Figure 5f), which corresponds to the diameter of silk nanofibrils reported previously.^57,58^ By contrast, the filaments formed after shearing were much thinner, having average height of ∼0.3 nm (Figure 5e). This height is comparable to the dimensions of main repetitive sequence of silk fibroin (GAGAS and GAGAGY), yet the length (>1 μm) is significantly longer than the chain length of native silk fibroin. This may indicate that the shear promoted the formation of intermolecular β-sheet through the main repetitive sequence of silk, connecting the multiple molecules and resulting in a micrometer-length filament. These filaments formed by shear presumably act as seeds and promoted the aggregation, as seen in the kinetic data of sheared samples (Figures 2 and 3). The thicker fibrils ∼4 nm (Figure 5f) are then presumably a bundle of nanofibrillar filaments that have come together.

## Conclusions

Silk is a material that has a plethora of biotechnological applications, most of which revolve around the self-assembly of soluble monomer to insoluble fibrils, which can be molded into different nanoarchitectures. Regenerated silk fibroin (RSF) is widely used for artificial functional silk-based materials, which possess remarkable properties. However, the mechanism by which monomers of this protein self-assemble into nanofibrils still remains largely unknown. Here, we provide a nano- and microscale understanding into the fibrillization of regenerated silk fibroin using a chemical kinetics approach. We determine that the nonclassical secondary pathway is the dominant process involved and that the presence of preformed seeds augments protein aggregation (Figure 1). Moreover, by employing a similar strategy, the effect of shear on the aggregation of RSF was determined. Our results suggest that the shear flow induces the formation of seeds, which catalyzes the formation of new fibrils through the secondary process (Figures 2 and 3). In addition, it was determined that the seed formation was enhanced when either the shear rate or the duration of shear was increased, indicating that both parameters contribute to the formation of primary nuclei. Finally, the morphology of both shear- and nonshear-formed aggregates were investigated using AFM (Figure 5). It revealed that the seeds formed by shear (∼0.3 nm thickness) are much thinner than mature fibrils (∼4 nm thickness). The presented kinetic approach allowed for the investigation of the nanoscale mechanism behind regenerated silk fibroin aggregation and the elucidation of the effect of hydrodynamic shear on the RSF self-assembly. Taken together, these results lead to a mechanistic description of the molecular-level processes governing RSF aggregation and forward the exploration of new possibilities for the development of next-generation bioinspired protein-based materials.

## Supporting Information Available

Aggregation kinetics of a 0.5 mg/mL regenerated silk fibroin solution after shearing for different time lengths; aggregation kinetics of bovine insulin with and without shear; FTIR spectra of regenerated silk fibroin before, after shearing, and following incubation at 37 °C for 72 h (PDF)

## References

[ref1] Hu X.; Cebe P.; Weiss A. S.; Omenetto F.; Kaplan D. L. Protein-based composite materials. Mater. Today 2012, 15, 208–215. 10.1016/S1369-7021(12)70091-3.

[ref2] Holland C.; Numata K.; Rnjak-Kovacina J.; Seib F. P. The biomedical use of silk: past, present, future. Adv. Healthcare Mater. 2019, 8, 1800465–1800491. 10.1002/adhm.201800465. 30238637

[ref3] Vollrath F.; Porter D. Silks as ancient models for modern polymers. Polymer 2009, 50, 5623–5632. 10.1016/j.polymer.2009.09.068.

[ref4] Koeppel A.; Holland C. Progress and Trends in Artificial Silk Spinning: A Systematic Review. ACS Biomater. Sci. Eng. 2017, 3, 226–237. 10.1021/acsbiomaterials.6b00669. 33465923

[ref5] Toprakcioglu Z.; Knowles T. P. Shear-mediated Sol-Gel Transition of Regenerated Silk Allows the Formation of Janus-like Microgels. Sci. Rep. 2021, 11, 6673 10.1038/s41598-021-85199-1. 33758259 PMC7988050

[ref6] Omenetto F. G.; Kaplan D. L. New opportunities for an ancient material. Science 2010, 329, 528–531. 10.1126/science.1188936. 20671180 PMC3136811

[ref7] Xu H.; Othman S. F.; Magin R. L. Monitoring tissue engineering using magnetic resonance imaging. J. Biosci. Bioeng. 2008, 106, 515–527. 10.1263/jbb.106.515. 19134545

[ref8] Kim S.; Marelli B.; Brenckle M. A.; et al. All-water-based electron-beam lithography using silk as a resist. Nat. Nanotechnol. 2014, 9, 306–310. 10.1038/nnano.2014.47. 24658173

[ref9] Marelli B.; Patel N.; Duggan T.; et al. Programming function into mechanical forms by directed assembly of silk bulk materials. Proc. Natl. Acad. Sci. 2017, 114, 451–456. 10.1073/pnas.1612063114. 28028213 PMC5255612

[ref10] Nguyen A. T.; Huang Q. L.; Yang Z.; et al. Crystal networks in silk fibrous materials: from hierarchical structure to ultra performance. Small 2015, 11, 1039–1054. 10.1002/smll.201402985. 25510895

[ref11] Shao Z.; Vollrath F. Surprising strength of silkworm silk. Nature 2002, 418, 741 10.1038/418741a. 12181556

[ref12] Heim M.; Keerl D.; Scheibel T. Spider silk: From soluble protein to extraordinary fiber. Angew. Chem., Int. Ed. 2009, 48, 3584–3596. 10.1002/anie.200803341. 19212993

[ref13] Rising A.; Johansson J. Toward spinning artificial spider silk. Nat. Chem. Biol. 2015, 11, 309–315. 10.1038/nchembio.1789. 25885958

[ref14] Sparkes J.; Holland C. Analysis of the pressure requirements for silk spinning reveals a pultrusion dominated process. Nat. Commun. 2017, 8, 594 10.1038/s41467-017-00409-7. 28928362 PMC5605702

[ref15] Asakura T.; Umemura K.; Nakazawa Y.; et al. Some observations on the structure and function of the spinning apparatus in the silkworm *Bombyx mori*. Biomacromolecules 2007, 8, 175–181. 10.1021/bm060874z. 17206804

[ref16] Gong Z.; Huang L.; Yang Y.; Chen X.; Shao Z. Two distinct β-sheet fibrils from silk protein. Chem. Commun. 2009, 17, 7506–7508. 10.1039/b914218e. 20024261

[ref17] Jin H. J.; Kaplan D. L. Mechanism of silk processing in insects and spiders. Nature 2003, 424, 1057–1061. 10.1038/nature01809. 12944968

[ref18] Kim U. J.; Park J.; Li C.; et al. Structure and properties of silk hydrogels. Biomacromolecules 2004, 5, 786–792. 10.1021/bm0345460. 15132662

[ref19] Matsumoto A.; Chen J.; Collette A. L.; et al. Mechanisms of silk fibroin sol-gel transitions. J. Phys. Chem. B 2006, 110, 21630–21638. 10.1021/jp056350v. 17064118

[ref20] Holland C.; Hawkins N.; Frydrych M.; et al. Differential Scanning Calorimetry of Native Silk Feedstock. Macromol. Biosci. 2019, 19, 6–11. 10.1002/mabi.201800228. 30411857

[ref21] Yucel T.; Cebe P.; Kaplan D. L. Vortex-induced injectable silk fibroin hydrogels. Biophys. J. 2009, 97, 2044–2050. 10.1016/j.bpj.2009.07.028. 19804736 PMC2756352

[ref22] Wang X.; Kluge J. A.; Leisk G. G.; Kaplan D. L. Sonication-induced gelation of silk fibroin for cell encapsulation. Biomaterials 2008, 29, 1054–1064. 10.1016/j.biomaterials.2007.11.003. 18031805 PMC2693043

[ref23] Greving I.; Cai M.; Vollrath F.; Schniepp H. C. Shear-induced self-assembly of native silk proteins into fibrils studied by atomic force microscopy. Biomacromolecules 2012, 13, 676–682. 10.1021/bm201509b. 22352290

[ref24] Shimanovich U.; Ruggeri F. S.; De Genst E.; et al. Silk micrococoons for protein stabilisation and molecular encapsulation. Nat. Commun. 2017, 8, 15902 10.1038/ncomms15902. 28722016 PMC5524934

[ref25] Sparkes J.; Holland C. The Energy Requirements for Flow-Induced Solidification of Silk. Macromol. Biosci. 2019, 19, 1–6. 10.1002/mabi.201800229. 30207051

[ref26] Laity P. R.; Holland C. The rheology behind stress-induced solidification in native silk feedstocks. Int. J. Mol. Sci. 2016, 17, 1812–1832. 10.3390/ijms17111812. 27801879 PMC5133813

[ref27] Holland C.; Terry A. E.; Porter D.; Vollrath F. Comparing the rheology of native spider and silkworm spinning dope. Nat. Mater. 2006, 5, 870–874. 10.1038/nmat1762. 17057700

[ref28] Laity P. R.; Gilks S. E.; Holland C. Rheological behaviour of native silk feedstocks. Polymer 2015, 67, 28–39. 10.1016/j.polymer.2015.04.049.

[ref29] Laity P. R.; Holland C. Native Silk Feedstock as a Model Biopolymer: A Rheological Perspective. Biomacromolecules 2016, 17, 2662–2671. 10.1021/acs.biomac.6b00709. 27315508

[ref30] Jin Y.; Hang Y.; Peng Q.; et al. Influence of shear on the structures and properties of regenerated silk fibroin aqueous solutions. RSC Adv. 2015, 5, 62936–62940. 10.1039/C5RA12885D.

[ref31] Koeppel A.; Laity P. R.; Holland C. Extensional flow behaviour and spinnability of native silk. Soft Matter 2018, 14, 8838–8845. 10.1039/C8SM01199K. 30349916

[ref32] Boulet-audet M.; Terry A. E.; Vollrath F.; Holland C. Acta Biomaterialia Silk protein aggregation kinetics revealed by Rheo-IR. Acta Biomater. 2014, 10, 776–784. 10.1016/j.actbio.2013.10.032. 24200713

[ref33] Li G.; et al. The natural silk spinning process: A nucleation-dependent aggregation mechanism?. Eur. J. Biochem. 2001, 268, 6600–6606. 10.1046/j.0014-2956.2001.02614.x. 11737214

[ref34] Dicko C.; Kenney J. M.; Knight D.; Vollrath F. Transition to a β-sheet-rich structure in spidroin in vitro: the effects of pH and cations. Biochemistry 2004, 43, 14080–14087. 10.1021/bi0483413. 15518557

[ref35] Dicko C.; Knight D.; Kenney J. M.; Vollrath F. Conformational polymorphism, stability and aggregation in spider dragline silks proteins. Int. J. Biol. Macromol. 2005, 36, 215–224. 10.1016/j.ijbiomac.2005.06.004. 16102807

[ref36] Dicko C.; Knight D.; Kenney J. M.; Vollrath F. Structural conformation of spidroin in solution: a synchrotron radiation circular dichroism study. Biomacromolecules 2004, 5, 758–767. 10.1021/bm034373e. 15132658

[ref37] Chen X.; Cai H.; Ling S.; Shao Z.; Huang Y. Conformation Transition of *Bombyx mori* Silk Protein Monitored by Time-Dependent Fourier Transform Infrared (FT-IR) Spectroscopy: Effect of Organic Solvent. Appl. Spectrosc. 2012, 66, 696–699. 10.1366/11-06551. 22732542

[ref38] Chen X.; Knight D. P.; Shao Z.; Vollrath F. Conformation Transition in Silk Protein Films Monitored by Time-Resolved Fourier Transform Infrared Spectroscopy: Effect of Potassium Ions on Nephila Spidroin Films. Biochemistry 2002, 41, 14944–14950. 10.1021/bi026550m. 12475243

[ref39] Zhong J.; et al. Self-assembly of regenerated silk fibroin from random coil nanostructures to antiparallel β-sheet nanostructures. Biopolymers 2014, 101, 1181–1192. 10.1002/bip.22532. 25088327

[ref40] Ling S.; Dinjaski N.; Ebrahimi D.; et al. Conformation Transitions of Recombinant Spidroins via Integration of Time-Resolved FTIR Spectroscopy and Molecular Dynamic Simulation. ACS Biomater. Sci. Eng. 2016, 2, 1298–1308. 10.1021/acsbiomaterials.6b00234. 33434983

[ref41] Rössle M.; Panine P.; Urban V. S.; Riekel C. Structural evolution of regenerated silk fibroin under shear: Combined wide- and small-angle x-ray scattering experiments using synchrotron radiation. Biopolymers 2004, 74, 316–327. 10.1002/bip.20083. 15211500

[ref42] Toprakcioglu Z.; Challa P.; Xu C.; P J Knowles T. Label-Free Analysis of Protein Aggregation and Phase Behavior. ACS Nano 2019, 13, 13940–13948. 10.1021/acsnano.9b05552. 31738513

[ref43] Knowles T. P. J.; Waudby C. A.; Devlin G. L.; et al. An analytical solution to the kinetics of breakable filament assembly. Science 2009, 326, 1533–1537. 10.1126/science.1178250. 20007899

[ref44] Knowles T. P. J.; Vendruscolo M.; Dobson C. M. The physical basis of protein misfolding disorders. Phys. Today 2015, 68, 36 10.1063/PT.3.2719.

[ref45] Meisl G.; Kirkegaard J. B.; Arosio P.; et al. Molecular mechanisms of protein aggregation from global fitting of kinetic models. Nat. Protoc. 2016, 11, 252–272. 10.1038/nprot.2016.010. 26741409

[ref46] Cohen S. I. A.; Vendruscolo M.; Welland M. E.; et al. Nucleated polymerization with secondary pathways. I. Time evolution of the principal moments. J. Chem. Phys. 2011, 135, 065105 10.1063/1.3608916. 21842954 PMC5017532

[ref47] Andreasen M.; Meisl G.; Taylor J. D.; et al. Physical determinants of amyloid assembly in biofilm formation. mBio 2019, 10, e02279-18 10.1128/mBio.02279-18. 30622185 PMC6325246

[ref48] Chengqian Y.; et al. Nucleation and Growth of Amino Acid and Peptide Supramolecular Polymers through Liquid–Liquid Phase Separation. Angew. Chem., Int. Ed. 2019, 58, 18116–18123. 10.1002/anie.201911782. 31617663

[ref49] Yuan C.; Ji W.; Xing R.; et al. Hierarchically oriented organization in supramolecular peptide crystals. Nat. Rev. Chem. 2019, 3, 567–588. 10.1038/s41570-019-0129-8. PMC7617026 39649433

[ref50] Törnquist M.; et al. Secondary nucleation in amyloid formation. Chem. Commun. 2018, 54, 8667–8684. 10.1039/C8CC02204F. 29978862

[ref51] Ferrone F. A.; Hofrichter J.; Eaton W. A. Kinetics of sickle hemoglobin polymerization: II. A double nucleation mechanism. J. Mol. Biol. 1985, 183, 611–631. 10.1016/0022-2836(85)90175-5. 4020873

[ref52] Cohen S. I. A.; et al. Proliferation of amyloid-β42 aggregates occurs through a secondary nucleation mechanism. Proc. Natl. Acad. Sci. 2013, 110, 9758–9763. 10.1073/pnas.1218402110. 23703910 PMC3683769

[ref53] Rockwood D. N.; Preda R. C.; Yücel T.; et al. Materials fabrication from *Bombyx mori* silk fibroin. Nat. Protoc. 2011, 6, 1612–1631. 10.1038/nprot.2011.379. 21959241 PMC3808976

[ref54] Gong Z.; Yang Y.; Huang L.; Chen X.; Shao Z. Formation kinetics and fractal characteristics of regenerated silk fibroin alcogel developed from nanofibrillar network. Soft Matter 2010, 6, 1217–1223. 10.1039/b913510c.

[ref55] Liu X.; Toprakcioglu Z.; Dear A. J.; et al. Fabrication and Characterization of Reconstituted Silk Microgels for the Storage and Release of Small Molecules. Macromol. Rapid Commun. 2019, 40, 1800898 10.1002/marc.201800898. 30840348

[ref56] Toprakcioglu Z.; Hakala T. A.; Levin A.; Becker C. F.; Bernardes G. G.; Knowles T. P. Multi-Scale Microporous Silica Microcapsules from Gas-in Water-in Oil Emulsions. Soft Matter 2020, 16, 3082–3087. 10.1039/C9SM02274K. 32140697

[ref57] Wang Q.; Ling S.; Yao Q.; et al. Observations of 3 nm Silk Nanofibrils Exfoliated from Natural Silkworm Silk Fibers. ACS Mater. Lett. 2020, 2, 153–160. 10.1021/acsmaterialslett.9b00461.

[ref58] Ling S.; Li C.; Adamcik J.; et al. Directed Growth of Silk Nanofibrils on Graphene and Their Hybrid Nanocomposites. ACS Macro Lett. 2014, 3, 146–152. 10.1021/mz400639y. 35590495

