# Supplementary material for: Kinetic Analysis Reveals the Role of Secondary Nucleation in Regenerated Silk Fibroin Self-Assembly
Source: Biomacromolecules. 2023 Mar 16;24(4):1709–16. doi: 10.1021/acs.biomac.2c01479 (PMC10091410; doi:10.1021/acs.biomac.2c01479)
Supplement: Supplementary file 1 — bm2c01479_si_001.pdf [file bm2c01479_si_001.pdf]

Supplementary information for

Kinetic Analysis Reveals the Role of Secondary Nucleation in Regenerated Silk Fibroin Self-Assembly

Ayaka Kamada[1,2†], Zenon Toprakcioglu[1†] and Tuomas P.J. Knowles[1,3*]

[1]Department of Chemistry, University of Cambridge, Lensfield Road, Cambridge CB2 1EW, UK

[2]Current address: Xampla Ltd, BioInnovation Building, 25 Cambridge Science Park Rd, Cambridge CB4 0FW

[3]Cavendish Laboratory, University of Cambridge, CB3 0FE, Cambridge UK

[*] Corresponding to: tpjk2@cam.ac.uk

†These authors contributed equally to this work

Supplementary Figures

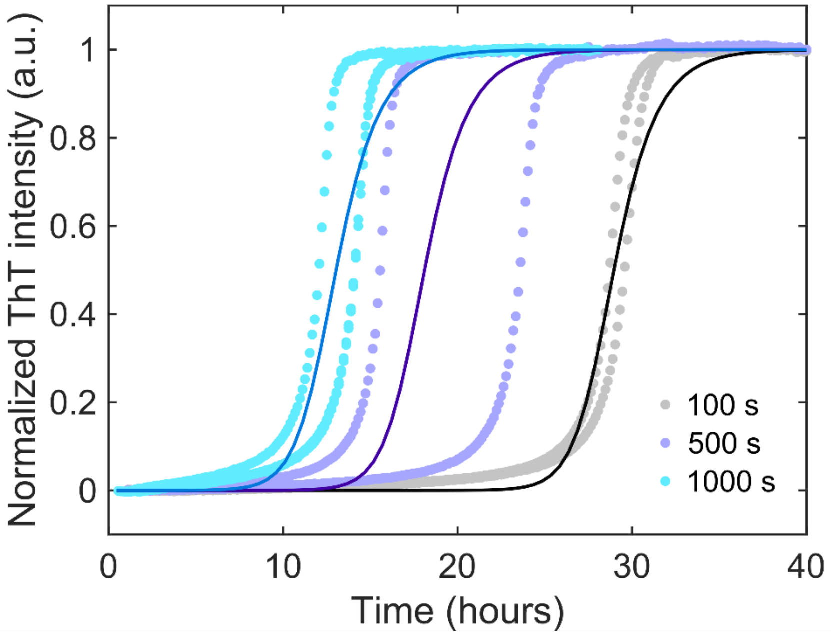

Supplementary Figure S1. ThT fluorescence intensity as a function of time for 0.5 mg/mL regenerated silk fibroin sheared at shear rate of 100 s^{-1}. The solid line indicated the fitting obtained from Amylofit using secondary nucleation dominant model.

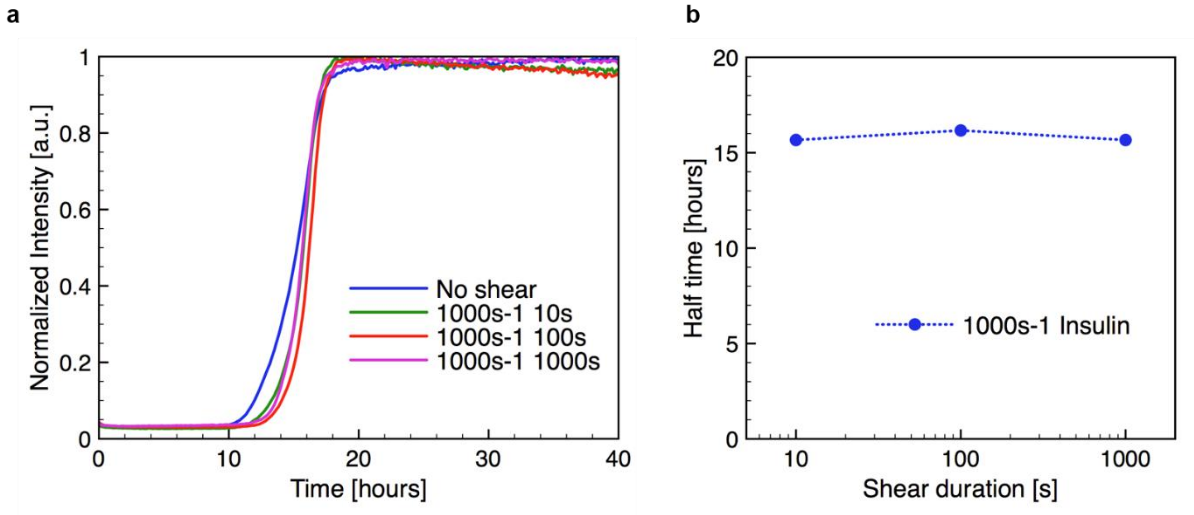

Supplementary Figure S2. Aggregation kinetics of sheared bovine insulin. (a) ThT fluorescence intensity as a function of time for insulin sheared with 1,000 s^{-1} for various duration, ranging from 10 s to 1000 s. (b) The half-time plot as a function of shearing time showing no significant effect of shear for insulin.

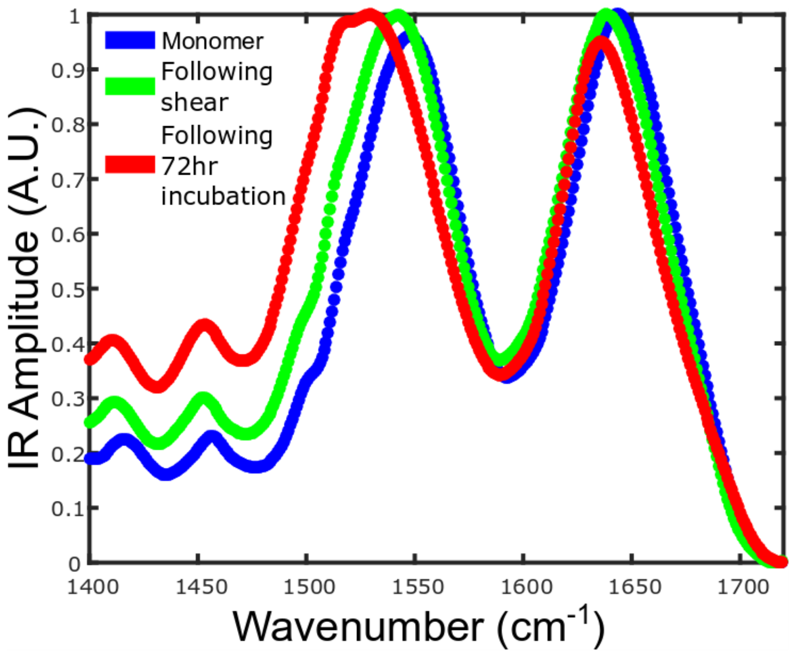

Supplementary Figure S2. FTIR spectra of regenerated silk fibroin in its monomeric state (blue curve), following shearing at a share rate of 500 s^{-1} for 100 s (green curve) and after a 72 hour incubation in order to promote protein self-assembly (red curve).
